# Dose–responses from multi-model inference for the non-cancer disease mortality of atomic bomb survivors

**DOI:** 10.1007/s00411-012-0410-4

**Published:** 2012-03-22

**Authors:** H. Schöllnberger, J. C. Kaiser, P. Jacob, L. Walsh

**Affiliations:** 1 Helmholtz Zentrum München, Department of Radiation Sciences, Institute of Radiation Protection, 85764 Neuherberg, Germany; 2 BfS-Federal Office for Radiation Protection, Neuherberg, Germany

**Keywords:** Risk assessment, Radiation, Cerebrovascular disease, Cardiovascular diseases, Threshold-dose, LNT

## Abstract

The non-cancer mortality data for cerebrovascular disease (CVD) and cardiovascular diseases from Report 13 on the atomic bomb survivors published by the Radiation Effects Research Foundation were analysed to investigate the dose–response for the influence of radiation on these detrimental health effects. Various parametric and categorical models (such as linear-no-threshold (LNT) and a number of threshold and step models) were analysed with a statistical selection protocol that rated the model description of the data. Instead of applying the usual approach of identifying one preferred model for each data set, a set of plausible models was applied, and a sub-set of non-nested models was identified that all fitted the data about equally well. Subsequently, this sub-set of non-nested models was used to perform multi-model inference (MMI), an innovative method of mathematically combining different models to allow risk estimates to be based on several plausible dose–response models rather than just relying on a single model of choice. This procedure thereby produces more reliable risk estimates based on a more comprehensive appraisal of model uncertainties. For CVD, MMI yielded a weak dose–response (with a risk estimate of about one-third of the LNT model) below a step at 0.6 Gy and a stronger dose–response at higher doses. The calculated risk estimates are consistent with zero risk below this threshold-dose. For mortalities related to cardiovascular diseases, an LNT-type dose–response was found with risk estimates consistent with zero risk below 2.2 Gy based on 90% confidence intervals. The MMI approach described here resolves a dilemma in practical radiation protection when one is forced to select between models with profoundly different dose–responses for risk estimates.

**Electronic supplementary material:**

The online version of this article (doi:10.1007/s00411-012-0410-4) contains supplementary material, which is available to authorized users.

## Introduction

One of the most important questions in radiation research relates to the shape of the dose–response for detrimental health effects at low doses, that is, whether any small dose of ionizing radiation adds to health risks, or whether there may be a threshold below which radiation may have no effect, or whether even protective effects may occur (Brenner et al. 2003; Averbeck 2009). This question bears essential relevance for our societies given, for example, the widespread use of medical imaging techniques such as CT scans, X-ray images, and mammography. It is also relevant for air crews and large worker populations who are exposed occupationally, for example, in nuclear installations. The possible risks of ionizing radiation are not limited to cancer but also relate to non-cancer diseases (Little et al. 2010). In that context, the question of a possible threshold or protective effects at low and/or medium doses is equally important as it is for cancer (Preston et al. 2003; Shimizu et al. 2010).

The mortality data from the Life Span Study (LSS), relating to the A-bomb survivors in Hiroshima and Nagasaki, are generally considered to be important for estimating the risk associated with ionizing radiation. Analyses of these data suggest a role of ionizing radiation in the formation of non-cancer diseases such as cerebrovascular disease (CVD)^1^ and cardiovascular diseases excluding CVD^2^ (Preston et al. 2003). Preston et al. (2003) concluded that the evidence for radiation effects on non-cancer mortality remains strong, with risks elevated by about 14% per Sv during the last 13 years of follow-up and that the best estimate for a threshold-dose is 0.2 Sv with an upper bound of about 0.7 Sv with no evidence against the linear-no-threshold hypothesis.

For protracted exposures, an important data set is the Mayak worker cohort (Azizova et al. 2008). The Mayak workers were exposed to low and medium doses at low dose rates. This together with the fact that these individuals did not have the threatening and traumatic experience of being exposed to the detonation of a nuclear bomb makes this data set especially valuable for risk estimations of general populations. Recently, statistically significant increasing trends in the incidence of cardiovascular and cerebrovascular diseases with external γ-ray dose have been reported for this cohort (Azizova et al. 2010a, b, 2011). Azizova et al. (2010a) found statistically significant increasing trends with both total external gamma-ray dose and internal liver dose in the incidence of ischaemic heart disease, a form of cardiovascular disease. They also reported statistically significant increasing trends in cerebrovascular disease incidence but not mortality with both total external γ-ray dose and internal liver dose from α-particle radiation (Azizova et al. 2010b, 2011).

In an extensive review, Little et al. (2010) present evidence for the epidemiological associations between lower-dose exposures and circulatory disease risks. They reviewed epidemiological data related to the atomic bomb survivors, low- and moderate-dose therapeutically exposed groups, and diagnostically, occupationally, and environmentally exposed groups. The authors conclude that the epidemiological evidence for an elevation of these diseases by moderate and low doses remains suggestive rather than persuasive (Little et al. 2010).

In the current study, various plausible dose–response curves (such as linear-no-threshold (LNT), linear quadratic, linear with threshold, step functions, hormesis-like dose–responses) were applied to the LSS data for CVD and cardiovascular diseases excluding CVD from Report 13 (Preston et al. 2003), and suitable quality-of-fit criteria were used to select the preferred models. A series of likelihood-ratio tests was used to obtain a set of preferable non-nested models. Multi-model inference (MMI), an innovative method to combine the estimates of several plausible non-nested models (Burnham and Anderson 2002; Claeskens and Hjort 2008), was then applied. The method resulted in a joint dose–response for each of the two biological endpoints. In the field of radiation epidemiology, MMI poses a fascinating new approach that avoids the danger of producing biased results from relying on just one single model of choice. Before the MMI method was introduced to radiation epidemiology by Walsh and Kaiser (2011), there was an earlier proposal to combine different probability distributions by assigning different probabilities to them regarding the possible existence of low-dose thresholds (Land 2002). This concept of Land (2002) can be regarded as a stimulating suggestion to apply MMI. For a further discussion of model selection criteria in radiation epidemiology, see the study by Walsh (2007).

An analysis of a more recent LSS data set with follow-up from 1950 to 2003 has also been performed (Shimizu et al. 2010). However, the question whether the dose–response is linear at low doses without threshold or whether nonlinear dose–response features are present is still unresolved. In the present study, it is shown that the shape of the dose–response curve cannot be found by exclusively using either the LNT or the linear threshold model, the approach used by Shimizu et al. (2010). The fact that several risk models yield plausible fits to the data is duly considered and accounted for here.

## Materials and methods

### Data on non-cancer disease mortality

The present analyses are based on two data sets for cerebrovascular disease (CVD; ICD-9 430–438) and cardiovascular diseases excluding CVD (ICD-9 390–429, 440–459) of LSS Report 13 (Preston et al. 2003; data file R13MORT.DAT from http://www.rerf.or.jp). In the remainder of this publication, the ICD-9 codes 390–429, 440–459 are simply referred to as cardiovascular diseases. In the file R13MORT.DAT, the data are provided in a person-year table and are categorized by city, sex, age at exposure, age attained, calendar time period during which the mortality checks were made, and weighted survivor colon dose. For each data group, the data file contains person-year weighted means of age attained, age at exposure, colon dose with a weight of ten for the neutron contribution, the number of person-years, and the number of deaths cases.

The data were analysed with exactly the same restrictions applied by Preston et al. (2003): we used data with follow-up starting on 1 January 1968 and ending on 31 December 1997. Only proximal survivors were taken where proximal is taken to mean survivors who were within a radius of 3 km from the hypocenter at the time of bombing. That gives 50,364 individuals (19,467 men and 30,897 women), of whom 3,954 died from CVD (1,434 men and 2,520 women) and 4,477 died from cardiovascular diseases (1,614 men and 2,863 women). The number of person-years is 1200,991.8 (452,161.6 and 748,830.2 person-years for men and women, respectively). Data pertaining to men and women were fitted jointly.

### Descriptive risk models

**Fig. 1:**
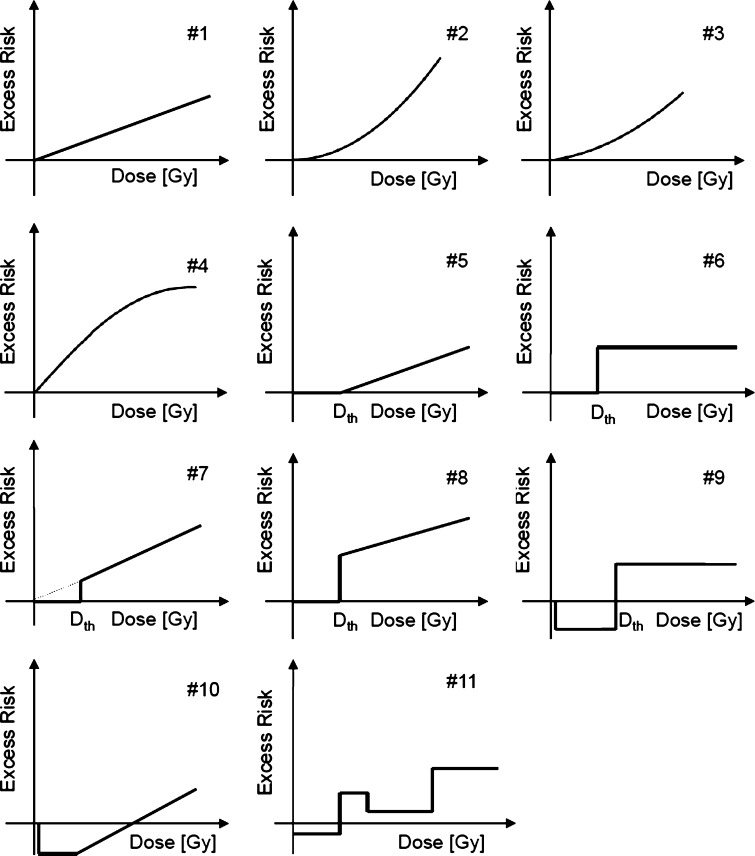
Parametric (#1 to #8, #10) and categorical (#9, #11) models used to investigate the shape of the dose–responses related to the risk that stems from ionizing radiation. 1st row: LNT model, quadratic model, linear-quadratic model; 2nd row: linear-exponential model, linear threshold model (sometimes only referred to as threshold model, the threshold-dose is denoted by *D*_*th*_), step model; 3rd row: step model with slope, another step model with slope, hormetic-like model; 4th row: hormetic-like model with slope; 3-step categorical model. Note that in both hormetic-like models the excess risk is set to zero for *D* < 0.005 Gy

The mortality data for CVD and cardiovascular diseases from Report 13 of the LSS were analysed with the following parametric and categorical models for the risk that stems from radiation: the LNT model, the quadratic model and the linear-quadratic model, the linear-exponential model, the linear threshold model (often referred to as threshold model within this study), various step models, hormesis-like models and one categorical model. Altogether, eleven different dose–responses were tested (Fig. 1). All of them were implemented either as excess relative risk (ERR) models or as excess absolute risk (EAR) models. The general form of an ERR model is as follows: *h* = *h*_0_ × (1 + *ERR*(*D*, *s*, *a*, *e*)) where *h* is the total hazard function, *h*_0_ is the baseline model and the function* ERR*(*D*, *s*, *a*, *e*) describes the change of the hazard function with weighted colon dose *D* allowing for effects of sex (*s*), age at exposure (*e*) and attained age (*a*). It is* ERR*(*D*, *s*, *a*, *e*) = *err*(*D*) × ε(*s*, *a*, *e*). Here,* err*(*D*) describes the shape of the dose–response function and ε(*s*, *a*, *e*) contains the dose-effect modifiers sex, age attained, and age at exposure. The general form of an EAR model is *h* = *h*_0_ + *EAR*(*D*, *s*, *a*, *e*) where* EAR*(*D*, *s*, *a*, *e*) = *ear*(*D*) × ε(*s*, *a*, *e*). Mathematical details related to the effect modifiers are given in Sect. 3 of the Online Resource. For *h*_0_, we first applied the Preston baseline model given in Eq. (A1) of the Online Resource (see file R13models.log at http://www.rerf.or.jp/library/dl_e/lss13.html, Preston et al. (2003)).

For* err*(*D*) and* ear*(*D*) the following dose–response models were used:

$$ {{err}}\left( D \right) = {{err}} \times D\quad {\text{LNT model}},\,\# 1 {\text{ in Fig}}.{ 1} $$

$$ {{err}}\left( D \right) = 1. 1 2 \times {{err}} \times D^{ 2} \quad {\text{Quadratic model}},\,\# 2 {\text{ in Fig}}.{ 1} $$

$$ {{err}}\left( D \right) = {{err}}_{ 1} \times D + 1. 1 2\times {{err}}_{ 2} \times D^{ 2} \quad {\text{Linear - quadr}}.{\text{ model}}, \, \# 3 {\text{ in Fig}}.{ 1} $$

$$ {{err}}\left( D \right) = \left( {{{err}}_{ 1} + {{err}}_{ 2} D} \right) \times { \exp }\left( { - {{err}}_{ 3} D^{ 2} } \right)\quad {\text{Linear - expon}}.{\text{ model}}, \, \# 4 {\text{ in Fig}}.{ 1} $$

$$ {{err}}(D) = \left\{ {\begin{array}{*{20}c} 0 & {D < D_{{th}} } \\ {{{err}}(D - D_{{th}} )} & {D \ge D_{{th}} } \\ \end{array} } \right\}\quad {\text{Linear thresh}}.{\text{ model}}, \, \# 5 {\text{ in Fig}}.{ 1} $$

$$ {{err}}\left( D \right) = 0. 5 \times {{scale}} \times \left[ {{ \tanh }\left( {s\left( {D - D_{{th}} } \right)} \right) + 1} \right]\quad {\text{Step model}}, \, \# 6 {\text{ in Fig}}.{ 1} $$

$$ err(D) = \left\{ {\begin{array}{*{20}c} 0 & {D < D_{{th}} } \\ {{{err}} \times D} & {D \ge D_{{th}} } \\ \end{array} } \right\}\quad {\text{Step model with slope}}, \, \# 7 {\text{ in Fig}}.{ 1} $$

$$ {{err}}(D) = \left\{ {\begin{array}{*{20}c} 0 & {D < D_{{th}} } \\ {{{err}}_{1} + {{err}}_{2} (D - D_{{th}} )} & {D \ge D_{{th}} } \\ \end{array} } \right\}\quad {\text{Step model with slope}}, \, \# 8 {\text{ in Fig}}.{ 1} $$

$$ {{err}}(D) = \left\{ {\begin{array}{*{20}c} 0 & {D < 0.005\,{\text{Gy}}} \\ {{{err}}_{1} } & {0.005\,{\text{Gy}} \le D < D_{{th}} } \\ {{{err}}_{2} } & {D \ge D_{{th}} } \\ \end{array} } \right\}\quad {\text{Hormesis - like model}}, \, \# 9 {\text{ in Fig}}.{ 1} $$

$$ {{err}}(D) = \left\{ {\begin{array}{*{20}c} 0 & {D < 0.005\,{\text{Gy}}} \\ {{{err}}_{1} } & {0.005\,{\text{Gy}} \le D < D_{{th}} } \\ {{{err}}_{1} + {{err}}_{2} (D - D_{{th}} )} & {D \ge D_{{th}} } \\ \end{array} } \right\}\quad {\text{Hormesis - like with}}\,{\text{slope}}, \, \# 10{\text{ in Fig}}.{ 1} $$

$$ {{err}}(D) = \left\{ {\begin{array}{*{20}c} {{{err}}_{1} } & {0 \le D < D_{1} } \\ {{{err}}_{2} } & {D_{1} \le D < D_{2} } \\ {{{err}}_{3} } & {D_{2} \le D < D_{3} } \\ {{{err}}_{4} } & {D \ge D_{3} } \\ \end{array} } \right\}\quad 3 {\text{ - step categorical model}}, \, \# 1 1 $$

The necessary adjustments for random errors in dosimetry applied to the dose term are already applied in the publicly available data, but a separate adjustment involving a multiplication factor to the dose-squared covariable should be done explicitly, either according to Pierce et al. (1990) (factor 1.12) or Pierce et al. (2008) (revised factor 1.15). Since most of the published analyses apply the factor 1.12, this has been adopted here for the quadratic and linear-quadratic models.

The Preston baseline model (given in Eq. (A1) of the Online Resource) was optimized here with series of likelihood-ratio tests. For nested models, the difference between their deviances (dev) is χ^2^-distributed (Claeskens and Hjort 2008). A model is considered an improvement over another model with a 95% probability if the deviance is lowered by at least 3.84 points after adding of one parameter. A description of this streamlining process, which has also been applied in a recent study on breast cancer risk in atomic bomb survivors (Kaiser et al. 2011), is given below.

### Streamlining the Preston baseline model

Preston’s fit to the LSS data for CVD (presented in Table 13 in Preston et al. (2003)) was reproduced in the first step. Preston et al. (2003) concluded that an LNT model implemented as ERR model fitted the data best. In order to reproduce this, the Preston baseline model given in Eq. (A1) of the Online Resource was combined with an LNT model, implemented as an ERR model and fitted to the joint data for CVD in men and women. This model is referred to as Preston’s ERR-LNT model and contains 30 model parameters (dev = 3599.58, Table 1). Then, each of the 29 baseline parameters was tested for its significance at the 95% significance level by setting it to 0 and refitting all the other parameters. Rigorous testing led to a new set of statistically significant baseline parameters, with eight parameters less than Preston et al. (2003) used within their baseline model [*h*_0_ from Eq. (A1)]: the new model no longer contained four age at exposure dependences, the related three age knots, and one age attained dependence. In addition, it was found that the model fit significantly improved when two other age knots and one age at exposure knot were allowed to be free (for details consult Sect. 2 of the Online Resource). The streamlined baseline model for CVD, which was used in combination with the 11 models depicted in Fig. 1, therefore has 21 (29 − 8) model parameters (see Table S1 in the Online Resource).

**Table 1:** For both biological endpoints, the preferable final non-nested models are shown with related final deviances (dev), difference in final deviances (Δdev) with respect to the model with the smallest deviance, number of model parameters (*N*_*par*_), AIC-values, difference in AIC-values (ΔAIC) with respect to the model with the smallest AIC-value, and Akaike weights

	dev	Δdev	*N*_*par*_	AIC	ΔAIC	Weight
CVD (ICD-9 430–429)						
ERR-LNT model [#1]	3569.51	3.46	22	3613.51	1.46	0.2628
ERR-quadratic model [#2]	3570.14	4.09	22	3614.14	2.09	0.1918
ERR-step model [#6], *D*_*th*_ = 0.62 Gy	3566.05	0	23	3612.05	0	0.5454
Preston’s ERR-LNT model	3599.58	33.53	30	3659.58	47.53	–
Cardiovascular diseases (390–429, 440–459)						
EAR-LNT model^a^ [#1]	3693.73	0	17	3727.73	0	0.3619
ERR-quadratic model^a^ [#2]	3694.05	0.32	17	3728.05	0.32	0.1918
EAR-threshold model [#5], *D*_*th*_ = 2.0 Gy	3695.0	1.27	17	3729.0	1.27	0.1379
EAR-step model [#6], *D*_*th*_ = 2.19 Gy	3695.66	1.93	17	3729.66	1.93	0.3084
Preston’s ERR-LNT model	3709.71	15.98	30	3769.71	41.98	–

As a comparison, the values are also shown for Preston’s ERR-LNT models. Note that for cerebrovascular disease the three preferable models are ERR models; for cardiovascular diseases, the four preferable non-nested models are EAR models. The numbers in brackets refer to the eleven dose–responses depicted in Fig. 1

^a^Contains an age-dependent dose-effect modifier

For cardiovascular diseases, an analogous procedure was applied. Preston’s best fit of the data for cardiovascular diseases was reproduced: the Preston baseline model given in Eq. (A1) of the Online Resource was combined with an LNT model, implemented as ERR model and fitted to the joint data for cardiovascular diseases. The results of fitting Preston’s ERR-LNT model are given in Table 1: dev = 3709.71 with 30 model parameters. Then, each of the 29 baseline parameters was tested for its significance resulting in a streamlined baseline model with 14 model parameters less than the Preston baseline model, which also lost its city dependence (see Table S2 in the Online Resource). The streamlined baseline model no longer contained four age at exposure dependences, three age attained dependences, and the related five age knots. Furthermore, it was found that the model fit significantly improved when two other age knots were allowed to be free (for details consult Sect. 2 of the Online Resource). The streamlined baseline model for cardiovascular diseases therefore has 15 (29 − 14) model parameters (see Table S2 in the Online Resource).

### Fitting the descriptive risk models

After having acquired two streamlined baseline models for CVD and cardiovascular diseases with the procedure described in the previous two paragraphs, all other models (i.e. models other than the LNT model that was already used for the streamlining process) depicted in Fig. 1 were also combined with the streamlined baseline models as either ERR model or EAR model and fitted to the data for CVD and cardiovascular diseases. For those parametric and categorical models that contain a threshold-dose *D*_*th*_, the following set of different values for *D*_*th*_ was used to carefully investigate which value leads to the smallest deviance: 0.0001 Gy, 0.0002,…, 0.0005, 0.001, 0.005, 0.01, 0.02, …, 0.09, 0.1, 0.2, …, 0.9, 1, and 2 Gy. In the linear threshold model, however, *D*_*th*_ was adjusted in the model fit. The step model was replaced by a modified hyperbolic tangent function as described below. Throughout this extensive approach, likelihood-ratio tests were applied to compare nested models with each other, to eliminate those nested models with inferior deviance values and to obtain two final sub-sets of non-nested models, one for each detrimental health outcome.

The step model (Fig. 1) was not implemented as a categorical model. Instead, the following modified hyperbolic tangent was used: 0.5 × *scale* × [tanh(*s*(*D* − *D*_*th*_)) + 1]. With appropriate values for *scale*, slope *s*, and *D*_*th*_, this flexible function can accommodate various entirely different shapes, among them the step function as depicted in Fig. 1 (model #3). With the hyperbolic tangent, steps are not imposed a priori but are a result of a fit to the data. The advantage of this function is the fact that it generally allows an estimate of *D*_*th*_ to be obtained with greater accuracy by fitting the model to data, while in a categorical implementation a value of *D*_*th*_ has to be assumed for each fit.

It was also successively investigated whether or not any of the three dose-effect modifiers, that is, sex, age attained, and age at exposure improved the model fits significantly.

### Data-fitting techniques and MMI

The MECAN software (Kaiser 2010) was applied to fit the EAR and ERR models to the data. This software uses Poisson regression (Schöllnberger et al. 2006) to estimate the values of the adjustable model parameters by fitting the model to the data. For the minimization of the Poisson deviance, MECAN applies Minuit2 (2008). Symmetric, Wald-type standard errors are calculated for the parameter estimates.

The* ERR* and* EAR* risk estimates are calculated directly from the hazard function:

1 $$  \begin{aligned}   {{ERR}} &  = (h/h_{0} ) - 1 \\    {{EAR}} &  = h - h_{0} . \\  \end{aligned}$$

Confidence intervals (CI) for the risk estimates given in Eq. (A1) are calculated with Latin hypercube sampling (LHS) which accounts for uncertainties and correlations of all adjustable parameters. For a risk variable such as* ERR*, a probability density distribution of 10^4^ realizations is generated, which is used to derive statistical descriptors such as mean, median, and percentiles. The MECAN software (Kaiser 2010) allows to perform Poisson regression, comparison of observed and expected cases, and simulation of uncertainty intervals within one run. The software package and all model-related input and result files are available from the authors upon request.

For both investigated detrimental health outcomes, the final non-nested models, which are presented in the “Results” section, were weighted according to the AIC (see below) and used to perform MMI, which is a method of mathematically superposing different non-nested models that all describe a certain data set almost equally well (Burnham and Anderson 2002). The method applies Akaike’s Information Criterion (Akaike 1973, 1974): AIC = dev + 2 *N*_par_, where *N*_par_ is the number of model parameters. For each model fit, an AIC-value is calculated. For a set of *n* non-nested models, the Akaike weight, *p*_*m*_, is calculated for model *m* according to the following equation (Claeskens and Hjort 2008):

2 $$ p_{m} = \frac{{\exp \left( { - \Updelta {\text{AIC}}_{m} /2} \right)}}{{\sum\nolimits_{j = 1}^{n} {\exp \left( { - \Updelta {\text{AIC}}_{j} /2} \right)} }} . $$

Here, ΔAIC_*m*_ = AIC_*m*_ − AIC_0_, where AIC_*m*_ is the AIC-value for model *m* and AIC_0_ is the smallest AIC-value of all *n* models. The resulting weights, multiplied by a factor of 10^4^, give the number of samples for risk estimates to be generated by LHS simulations. Then, for each set of preselected values of age attained, age at exposure, and dose, the created model-specific probability density functions (PDFs) are merged. The resulting probability density functions, each of size 10^4^, represent all uncertainties arising within a model and from the superposition of the selected models. Statistical quantities such as mean, median, and percentiles are derived from the final PDFs.

Below, larger deviances compared to our best models (i.e. those with smallest AIC-values) are denoted by positive values of Δdev. The notation Δpar gives the difference in number of parameters compared to the models with smallest AIC.

## Results

Using the approach outlined in the “Materials and methods” section, it was found that for CVD the following final three non-nested ERR models out-competed all other models and were included in the sub-set for MMI: an ERR-LNT model consisting of the streamlined baseline model with 21 significant baseline parameters combined with an LNT model via parameter* err* (Δdev = 3.46; Table 1), an ERR-quadratic model (Δdev = 4.09; Table 1), and an ERR-step model with *D*_*th*_ = 0.62 Gy (Δdev = 0; Table 1 and Fig. 1). Table 1 gives for these final three non-nested models all essential information obtained by fitting them to the CVD data. Table S1 in the Online Resource provides all related model parameters and related best estimates together with Wald-type standard errors: all three models contain 21 baseline parameters; the ERR-LNT model and the ERR-quadratic model each contain one radiation-related parameter (*err*); the ERR-step model has two radiation-related parameters (*scale*, *D*_*th*_). As a comparison, Table 1 also includes the results for Preston’s ERR-LNT model: Δdev = 33.53 and Δpar = 7, that is, even though Preston’s ERR-LNT model has 7 parameters more than our ERR-step model, the latter still leads to a better fit than the Preston model by 33.53 deviance points. This improvement in fit is related to the free age knots and age at exposure knots described in the “Materials and methods” section.

For cardiovascular diseases, the MMI sub-set consisted of four non-nested EAR models: an EAR-LNT model (Δdev = 0), an EAR-quadratic model (Δdev = 0.32), an EAR-threshold model with *D*_*th*_ = 2.0 Gy (Δdev = 1.27), and an EAR-step model with *D*_*th*_ = 2.19 Gy (Δdev = 1.93). The first two models both include a dose-effect modifier that depends on age attained. The step model was implemented as a hyperbolic tangent function. Table 1 gives, for each of the final four models, all essential information obtained by fitting them to the data for cardiovascular diseases. Refer to Table S2 (Online Resource) for all related model parameters (baseline and radiation related), their best estimates and Wald-type standard errors. It is noted that for younger ages the significant dose-effect modifier in the EAR-LNT model leads to smaller slopes than the one depicted in Fig. 3 (see Sect. 3 of the Online Resource for details). As a comparison, Table 1 also includes the results for Preston’s ERR-LNT model: Δdev = 15.98 and Δpar = 13, that is, although Preston’s ERR-LNT model has 13 parameters more than our EAR-LNT model, the latter fits the data for cardiovascular diseases by 15.98 deviance points better than Preston’s ERR-LNT model (Table 1).

**Fig. 2:**
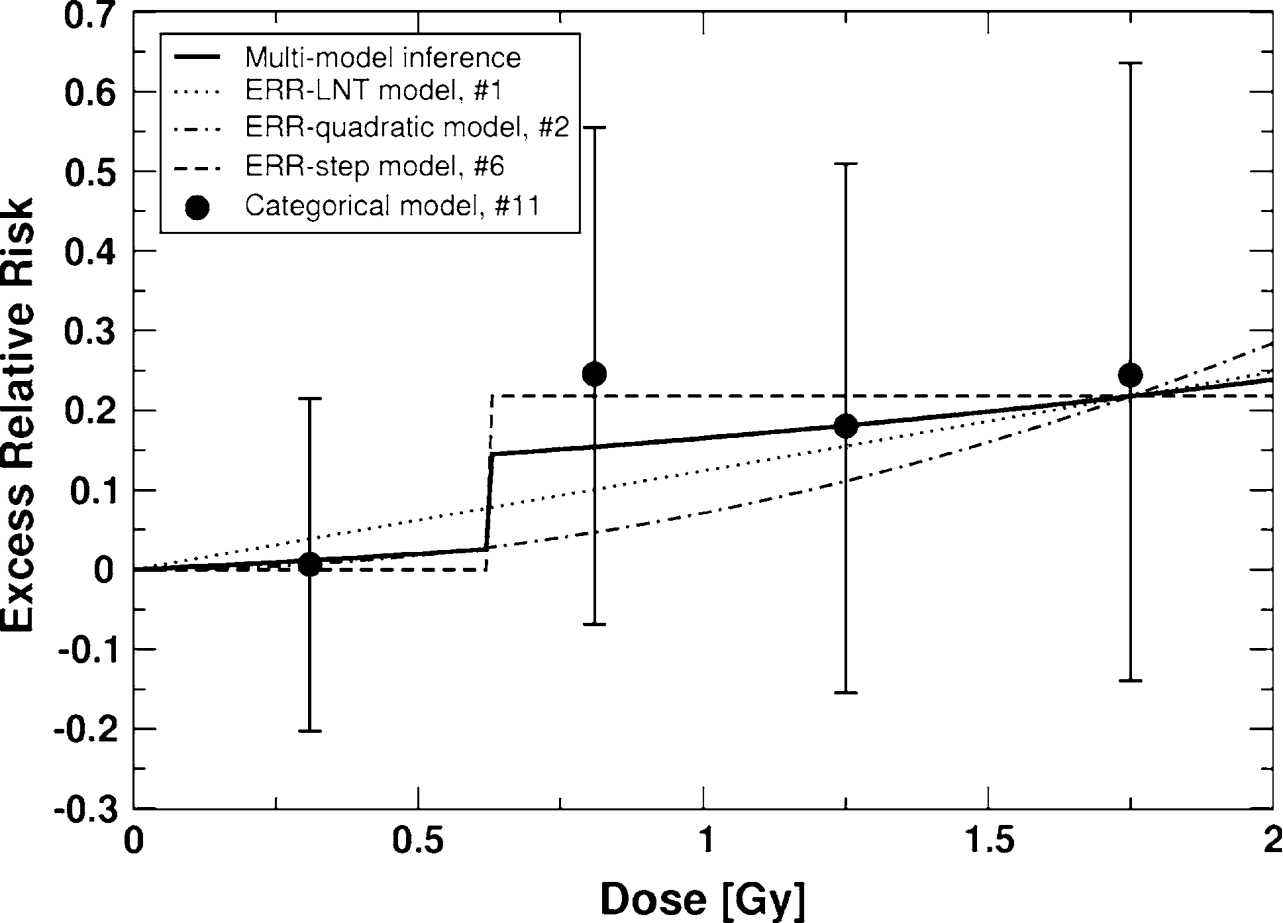
*ERR* for cerebrovascular disease versus weighted colon dose for the final three non-nested ERR models and the multi-model inference (MMI) (Table 1). Also shown are point estimates and related 90% CI for a 3-step categorical ERR model that divides the dose range into four categories: *D* < 0.62 Gy, 0.62 Gy ≤ *D* < 1 Gy, 1 Gy ≤ *D* < 1.5 Gy, and *D* ≥ 1.5 Gy. The 90% CI for the MMI are provided in Table 2 for absorbed doses of 0.2 and 1 Gy. The figure is valid for men and women of both cities. The preselected values for age at exposure and age attained are 30 and 70 years, respectively

**Fig. 3:**
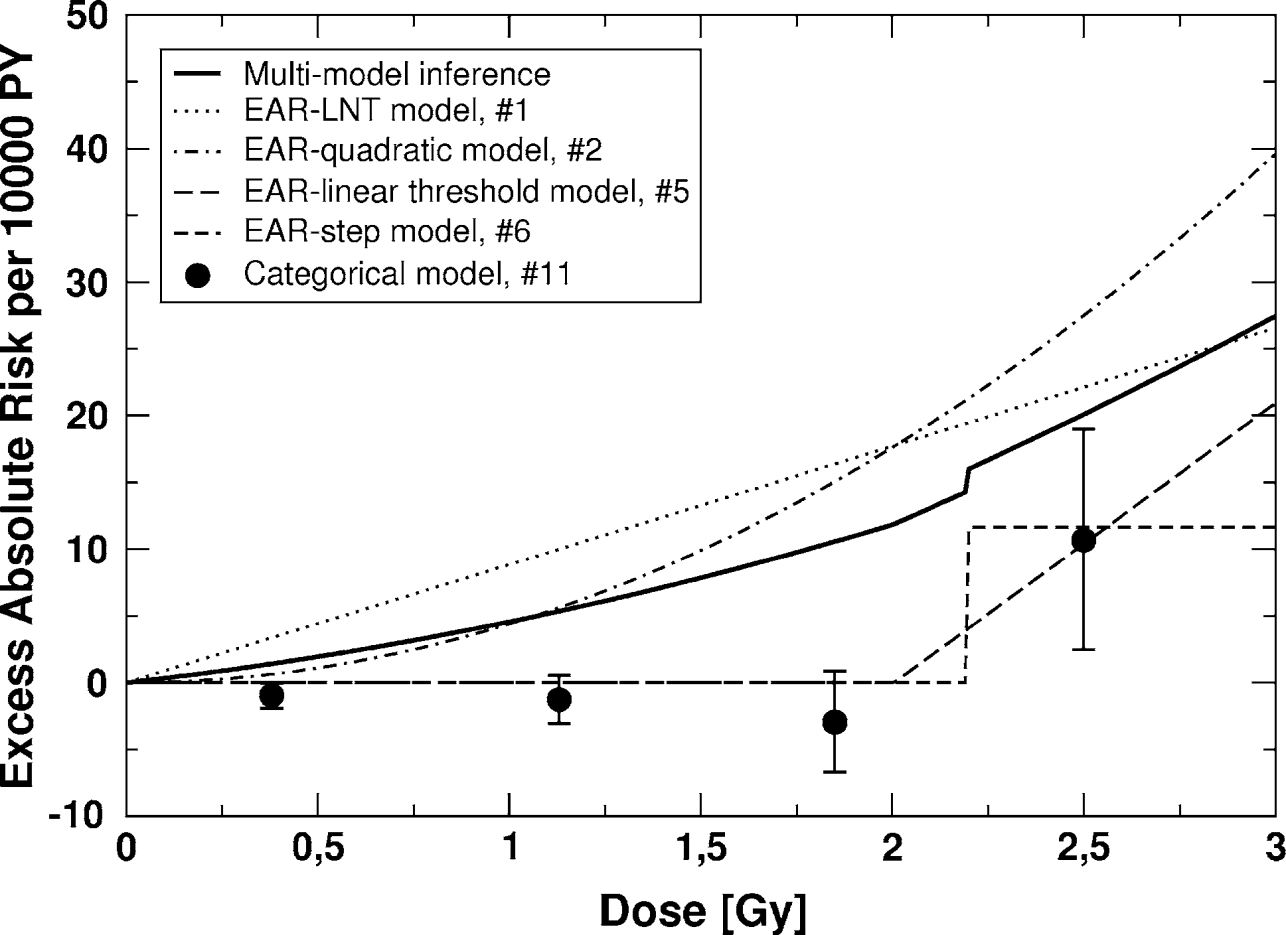
*EAR* for cardiovascular diseases versus weighted colon dose for the final four non-nested EAR models and the multi-model inference (refer to Table 1). Also shown are point estimates and related 90% CI for a 3-step categorical ERR model that divides the dose range into four categories: *D* < 0.75 Gy, 0.75 Gy ≤ *D* < 1.5 Gy, 1.5 Gy ≤ *D* < 2.19 Gy, and *D* ≥ 2.19 Gy. The 90% CI for the MMI are provided in Table 3 for an absorbed dose of 1 Gy. The figure is valid for men and women of both cities. The preselected values for age at exposure and age attained are 30 and 70 years, respectively

The related AIC-values are shown in Table 1 together with the Akaike weights *p*_*m*_ (2). The latter were used to perform MMI as described in the “Materials and methods” section. The results are shown in Figs. 2 and 3. For CVD, the deviance of 3566.57 (Δdev = 0.49) related to MMI is easily obtained, since the dose–response contains no dose–effect modifiers. The MMI predicts a very low* ERR* for doses below the threshold, because of the contribution from the ERR-step model with a threshold-dose of 0.62 Gy, and the 95% CIs include zero risk (Table 2). Therefore, the MMI risk estimates for CVD presented here are consistent with zero risk below the threshold of 0.62 Gy. The results for cardiovascular diseases follow a similar pattern: based on the 90% CI, the MMI implies zero risk up to 2.24 Gy.

**Table 2:** Values for* ERR* and* EAR* for cerebrovascular disease calculated with the multi-model inference, the ERR-LNT model, the ERR-quadratic model, and the ERR-step model for 0.2 and 1 Gy and different values of age at exposure (*e*) and age attained (*a*)

	*ERR*	*EAR* [per 10^4^ PY]
*CVD*		
Multi-model inference		
0.2 Gy		
*e* = 20, *a* = 50	0.007 (0, 0.035)	0.05 (0, 0.23)
*e* = 20, *a* = 70	0.007 (0, 0.035)	0.17 (0, 0.84)
*e* = 30, *a* = 70	0.007 (0, 0.035)	0.3 (0, 1.4)
*e* = 50, *a* = 70	0.007 (0, 0.035)	0.8 (0, 3.8)
1 Gy		
*e* = 20, *a* = 50	0.165 (0.033, 0.32)	1.10 (0.22, 2.1)
*e* = 20, *a* = 70	0.165 (0.033, 0.32)	3.97 (0.78, 7.7)
*e* = 30, *a* = 70	0.165 (0.033, 0.32)	6.6 (1.3, 13)
*e* = 50, *a* = 70	0.165 (0.033, 0.32)	18.0 (3.6, 35)
Single models		
0.2 Gy, *e* = 30, *a* = 70		
ERR-LNT model [#1]	0.0248 (0.0055, 0.044)	0.98 (0.22, 1.7)
ERR-quadratic model [#2]	2.84 × 10^−3^ (4.0 × 10^−4^, 5.3 × 10^−3^)	0.114 (0.016, 0.21)
ERR-step model [#6], *D*_*th*_ = 0.62 Gy	0	0
1 Gy, *e* = 30, *a* = 70		
ERR-LNT model [#1]	0.124 (0.028, 0.22)	4.9 (1.1, 8.7)
ERR-quadratic model [#2]	0.071 (0.010, 0.13)	2.85 (0.40, 5.3)
ERR-step model [#6], *D*_*th*_ = 0.62 Gy	0.22 (0.093, 0.34)	8.7 (3.7, 14)
Preston ERR-LNT model (Preston et al. 2003)	0.12 (0.02, 0.22)	5.0^a^ (1.0, 8.9)
ERR-LNT model (Shimizu et al. 2010)	0.09 (0.01, 0.17)^b^	2.3 (0.4, 4.4)^b^

The 90% confidence intervals are provided. The risk values from Preston et al. (2003) and Shimizu et al. (2010) are also shown. The numbers in brackets refer to the eleven dose–responses depicted in Fig. 1. The* EAR*-values for MMI and for the single models #1, #2, and #6 are only valid for men in Hiroshima. The city-averaged* EAR*-values for men can be calculated by multiplication with a factor of 1.1 (see Sect. 6 of the Online Resource for mathematical details). The* EAR*-values for women can be calculated by multiplying with a factor of 0.6

^a^Not given by Preston et al. (2003); calculated from Preston’s ERR-LNT model

^b^This is the 95% CI

The striking improvements of the deviances presented here compared with those from Preston’s ERR-LNT fits (Table 1) were mainly achieved by streamlining the baseline models. Therefore, better matches of observed and predicted cases were expected mainly in the group of “unexposed” survivors (i.e. individuals with doses below 5 mGy). To test this assumption, it was investigated which categories of dose and age attained contribute most to the decrease in deviance, found here with the preferred models, when compared to Preston’s ERR-LNT fits. For CVD, the preferred model according to AIC is the ERR-step model, for cardiovascular diseases it is the EAR-LNT model (Table 1). Using the related best estimates from Tables S1 and S2, forward calculations were performed with the data sets stratified into several groups of weighted colon dose and age attained. For CVD in men, the strongest contribution of 8.3 points to the improvement in deviance stems from individuals in dose category 0.1 < *D* ≤ 0.5 Gy with ages attained of 40 years and higher. For women, the strongest contribution of 19.8 points is related to dose categories 0.005 < *D* ≤ 0.1 Gy and 0.5 < *D* ≤ 1 Gy with ages attained of 40 years and higher. For cardiovascular diseases, the strongest contribution of 12 points stems from women in dose categories 0.1 < *D* ≤ 0.5 Gy and 0.5 < *D* ≤ 1 Gy at ages of 60 and higher, while men hardly improve the final deviance compared to the fit with Preston’s ERR-LNT model (1915.21 versus 1915.88). Detailed results can be seen in Tables S3, S4, and S5 in the Online Resource.

For both detrimental health outcomes, the risk estimates* ERR* and* EAR* were calculated for the multi-model inferences and for the non-nested models listed in Table 1. The results are given in Tables 2 and 3 for a dose of 1 Gy and for different values of age attained (50 and 70 years) and age at exposure. For CVD and cardiovascular diseases, the mean age of the cases (i.e. of individuals who died from these diseases) was about 77 and 78 years, respectively. Because of the threshold at 0.62 Gy for CVD, for this disease* ERR* and* EAR* were also calculated for 0.2 Gy. The risk estimates from Preston et al. (2003) and Shimizu et al. (2010) are also provided. For CVD, the* EAR* depends on city and sex because it is calculated from ERR models and because the streamlined baseline model presented here depends on city and sex. Therefore, the* EAR*-values for MMI and for the single models #1, #2, and #6 in Table 2 are only valid for men from Hiroshima. For cardiovascular diseases, the* ERR* depends on sex because it is calculated from EAR models and because the applied streamlined baseline model depends on sex (details are given in Sect. 5 of the Online Resource).

**Table 3:** Values for* ERR* and* EAR* for cardiovascular diseases calculated with the multi-model inference, the EAR-LNT model, the EAR-quadratic model, the EAR-threshold model, and the EAR-step model for 1 Gy and different values of age at exposure (*e*) and age attained (*a*)

	*ERR*	*EAR* [per 10^4^ PY]
*Cardiovascular diseases*		
Multi-model inference		
*e* = 20, *a* = 50	0.10 (0, 0.35)	0.8 (0, 2.7)
*e* = 20, *a* = 70	0.12 (0, 0.35)	5 (0, 13)
*e* = 30, *a* = 70	0.09 (0, 0.25)	5 (0, 13)
*e* = 50, *a* = 70	0.07 (0, 0.18)	5 (0, 13)
Single models		
*e* = 30, *a* = 70		
EAR-LNT model [#1]	0.171 (0.078, 0.27)	8.8 (4.2, 14)
EAR-quadratic model [#2]	0.084 (0.026, 0.14)	4.4 (1.4, 7.4)
EAR-threshold model [#5], *D*_*th*_ = 2.0 Gy	0	0
EAR-step model [#6], *D*_*th*_ = 2.19 Gy	0	0
Preston’s ERR-LNT model, Preston et al. (2003)	0.17 (0.08, 0.26)	9.1^a^ (4.2, 13.9)
ERR-LNT model, Shimizu et al. (2010)	0.14 (0.06, 0.23)^b^	3.2 (1.3, 5.2)^b^

The 90% confidence intervals are provided. The risk values from Preston et al. (2003) and Shimizu et al. (2010) are also shown. The numbers in brackets refer to the eleven dose–responses depicted in Fig. 1. The ERR-values for MMI and for the single models #1, 2, 5, and #6 are only valid for men. The ERR-values for women can be calculated by multiplication with a factor of 1.8

^a^Not given by Preston et al. (2003); calculated from Preston’s ERR-LNT model

^b^This is the 95% CI

## Discussion

In the present study, the dose–responses of the LSS non-cancer mortality data for CVD and cardiovascular diseases were investigated using different parametric and categorical models (Fig. 1). Two sub-sets of final, preferable, non-nested models were identified, one for each detrimental health outcome. These models are summarized in Table 1. They all describe the data about equally well: only relatively small differences in deviances and AIC-values were found.

For CVD, the ERR-step model (model #6 in Fig. 1; with the step smoothed by the hyperbolic tangent function) with a threshold-dose of *D*_*th*_ = 0.62 Gy has the lowest AIC. The LNT model and the quadratic model are also included in the MMI (Fig. 2), resulting in a weak dose–response below the threshold (with a risk estimate of about one-third of that from the LNT model) and a stronger dose–response for higher doses. MMI results in a small excess relative risk below the threshold. The 90% confidence intervals are compatible with no risk up to 0.62 Gy (Table 2). This is confirmed by a fit using a categorical model: the risk estimate in the lowest dose group is not significantly different from zero (Fig. 2).

An analogous argument holds for the analysis of the LSS data for cardiovascular diseases (Fig. 3). Again, the MMI does not contain any threshold-dose but the lower bound of the related 90% CI at 1 Gy is zero (Table 3). The MMI is in fact consistent with zero risk up to 2.24 Gy. In that context, it is notable that a fit with a categorical model infers a *U*-shaped dose–response, that is, negative excess absolute risk in the lower-dose regimes with a statistically significant negative risk in the lowest dose group (Fig. 3). The increasing risk with attained age (via the age-dependent dose-effect modifier) produces a markedly higher risk in the EAR-LNT model with 94 excess cases in contrast to 9 cases in the EAR-threshold model and the EAR-step model, where the effect modifier was not statistically significant. Consequently, the dose–response curve from MMI also predicts a strongly reduced risk for death from cardiovascular diseases due to radiation. In the context of the results presented here, it is interesting to point out a recent low-dose study in which ApoE null mice were used. This mouse model system spontaneously develops atherosclerosis when fed a normal low-fat diet. In these mice, the effects of single doses of 25–500 mGy, given at either early or late stage disease, were distinctly nonlinear with dose and were generally protective for various measures of the disease. In that animal model, most effects occurred below about 100 mGy, and many of the endpoints measured showed maximum protective effects at 25-50 mGy (Mitchel et al. 2011).

Related to Fig. 3, the* EAR* risk estimates for the EAR-LNT model, the EAR-quadratic model and for the MMI seem to be inconsistent with those calculated for the categorical fit, especially at the lower three doses. It is emphasized that this seeming inconsistency stems from the significant dose-effect modifier in the EAR-LNT model and the EAR-quadratic model (see Table S2 in the Online Resource). Figure 3 relates to an age attained of 70 years. For lower ages, the* EAR*-values for the EAR-LNT model are markedly decreased (numerical details are provided in Sect. 3 of the Online Resource). Consequently, this reduction also decreases the* EAR*-values for the MMI.

It is noted that for both diseases the categorical model (#11 in Fig. 1), a non-nested model, was not used for MMI because of its negligible contributions to the AIC-weights (Walsh 2007, Hoeting et al. 1999). Because of its similarity to the shape implied by the categorical model fit (Fig. 3), we also used the Gompertz curve to fit the excess absolute risk associated with the data for cardiovascular diseases. Again, it was found that the ΔAIC-based weight was too small to be used for MMI. For details, see Sect. 7 of the Online Resource.

Because of the well-known gender differences in cardiovascular disease mortality (Roger et al. 2011), it was investigated whether the data for men and women needed to be fitted separately. Model fits of the data for men and women were performed using an ERR-LNT model. For CVD, some differences were noted for the slope parameters (*err* = 0.109044 Gy^−1^ for men versus* err* = 0.13524 Gy^−1^ for women). However, comparing the related final deviances with the one from the joint fit (Table 1: dev = 3569.51 using 22 parameters) clearly showed that fitting the data for men and women separately does not lead to a significantly improved fit (men: dev = 1779.58 using 11 parameters; women: dev = 1788.24 using 13 parameters; sum = 3567.82). A similar result was found for cardiovascular diseases.

Preston et al. (2003) based their study on the use of the following five models: an LNT model, a linear-quadratic and a purely quadratic model, a linear threshold model, and categorical models implemented as either ERR model or EAR model. While Preston et al. (2003) report that there is no direct evidence of radiation effects for doses less than about 0.5 Sv, they conclude that radiation effects on LSS non-cancer mortality can be adequately described by a linear dose–response model. A data set on circulatory disease mortality with 6 years of additional follow-up has been publicly available since the end of 2010. Those data were analysed recently by Shimizu et al. (2010) with the LNT model and the linear threshold model (model #5 in Fig. 1) for a wide range of possible values of threshold-dose *D*_*th*_. They used differences in maximum likelihood to compare nested models and the AIC for non-nested models. For CVD, they report that the best estimate of a threshold-dose was 0.5 Gy but that this value was not statistically significant so that no threshold-dose may exist. For cardiovascular diseases, their best estimate of a threshold-dose was 0 Gy (Shimizu et al. 2010). In the present study, the earlier studies have been extended by using several additional possible dose–responses and by combining the results to obtain dose–responses and uncertainty ranges that are not based on assumptions made in a single model.

In their previous study, Preston et al. (2003) carefully explain why they did not use the full available data with follow-up starting in 1950. They state that characterization of the dose–response is complicated by a healthy survivor selection effect on non-cancer disease death rates. For a few years after the bombings, baseline (zero dose) non-cancer disease death rates for proximal survivors were markedly lower than those for distal survivors. The difference diminished steadily over the first two decades of follow-up, by which time it had largely vanished. This statistically significant pattern suggests that proximal survivors included in the LSS were initially healthier than the general population for reasons related to their selection by having survived the bombings. Analyses of the LSS non-cancer mortality data indicate that in 1950 baseline death rates for proximal survivors were 15% lower than those for distal survivors. The difference decreased to about 2% in the late 1960s (Preston et al. 2003). It has been illustrated by Preston et al. (2003) that a substantial healthy survivor selection leads to spurious curvature in the dose–response. According to Preston et al. (2003), the healthy survivor effect can be dealt with by restricting the analyses to proximal survivors and to the later period of follow-up, that is, 1968–1997. Unfortunately, the latest analysis of the LSS non-cancer data was done for the full cohort and for the full period of follow-up, that is, 1950–2003 (Shimizu et al. 2010). Concern related to the fact that Shimizu et al. (2010) place completely different emphasis and importance on the reported magnitude of the healthy survivor bias has been raised by Walsh (2011). Note that the downloadable grouped data by Shimizu et al. (2010) do not contain the same grouping boundaries as the data used in the present study: there is no proximal/distal group and no boundary corresponding to follow-up starting on 1 January 1968. A preliminary analysis of exactly the same mortality data for CVD that Shimizu et al. (2010) used (i.e. follow-up 1950–2003) using a streamlined Preston baseline model showed that an ERR-LNT model is preferable. It is interesting to note that when analysing the Shimizu CVD data for the follow-up 1971-2003 (and thereby including most of the original Preston et al. 2003 data plus the additional 6 years of follow-up plus the distal survivors), the present authors found confirmation for the threshold-dose of 0.6 Gy obtained in the current study. The Shimizu CVD data for the follow-up 1971–2003 were analysed in the same way as the Preston et al. (2003) data. The Preston baseline model [Eq. (A1) of the Online Resource] was combined with an ERR-LNT model and fit to the data for CVD. The Preston baseline model was then streamlined using the likelihood-ratio test and then combined with the step model from Fig. 1 as an ERR model. The related best estimates and Wald-type standard errors (in parenthesis) are as follows: *D*_*th*_ = 0.64 Gy (< 1%), *scale* = 0.204 (0.081) with a fixed value for the slope *s*: 10^5^/Gy (compare with Table 1 in the Online Resource). However, because of the above-mentioned incompatibility of the Shimizu et al. (2010) data with the data used by Preston et al. (2003), the analysis of the publicly available data set was not continued. Instead, the present authors are planning to pursue the analysis of a more suitable data set with a time cut-point at 1 January 1968 and an added indicator to distinguish proximal from distal survivors to be created by the Radiation Effects Research Foundation (RERF) in Japan.

Application of the AIC criterion for model selection exacts a rigorous application of parameter parsimony, since model weights are very sensitive to differences in AIC. The authors do not claim to have identified the optimal models. There is a potential to detect better parameterizations by fitting nonparametric models to the baseline death rates. However, the introduction of nonparametric baseline models into MMI requires further theoretical investigations by a larger number of experts. The present study leads to streamlined fully parametric baseline models (with significantly lower deviances despite the smaller number of model parameters) compared to the Preston baseline model (Preston et al. 2003). However, the risk estimates presented here with LNT models almost exactly correspond to those of Preston et al. (2003) (Tables 2 and 3).

In addition to these observed threshold-doses, another important difference from the earlier work of Preston et al. (2003) and Shimizu et al. (2010) is that the analyses presented here for the radiation influence on cardiovascular diseases actually favour EAR-risk models. The other authors prefer ERR models but renounced the rigorous application of quality-of-fit criteria.

In a review of published low-/moderate-dose epidemiological data sets on circulatory diseases, Little et al. (2010) list in their Table 1 14 studies related to the following exposed populations: atomic bomb survivors, low- and moderate-dose therapeutically exposed groups, diagnostically exposed groups, occupationally and environmentally exposed groups. Here, the dose–response models applied in these 14 studies are briefly reviewed. The two papers analysing LSS non-cancer data are by Preston et al. (2003) and Yamada et al. (2004). The study of Preston et al. (2003) made use of four different dose–response models and has already been summarized above. Yamada et al. (2004) assumed an additive linear dose–response model: *RR*_*ij*_ = 1 + β*d*_*ij*_ exp(α_*k*_(*Z*_*k*_)), where *RR*_*ij*_ is the relative risk due to radiation dose associated with the *j*th exposure level, *d*_*ij*_ is the *j*th dose level in stratum *i,* β is the excess risk per Sievert averaged over all strata, and *Z*_*k*_ represents the effect modifiers (Yamada et al. 2004). They also tested linear-quadratic and purely quadratic models. For circulatory disease-related endpoints, such as hypertension, ischaemic heart disease, myocardial infarction and stroke, Yamada et al. (2004) did not find a statistically significant dependence on radiation exposure. Little et al. (2010) additionally included the following three studies related to low-dose radiotherapy and medical diagnostics. Carr et al. (2005) fitted a generalized linear model to a cohort of 3,719 peptic ulcer disease patients treated with radiotherapy or by other means. In the studies by Darby et al. (1987) and Davis et al. (1989), the standardized mortality ratio (SMR; number of observed cases divided by number of expected) as a precursor to modelling dose–response curves was calculated. The following eight occupational studies were also reviewed by Little et al. (2010). Ashmore and colleagues analysed the mortality from cancer and non-cancer diseases within a large cohort of Canadian radiation workers comprising 206,620 individuals. They used a relative risk model with risk increasing linearly with dose (Ashmore et al. 1998). Azizova and Muirhead (2009) modelled the ERR in the Mayak worker cohort by a linear trend with external or internal dose. In their analysis of 61,017 Chernobyl emergency workers, Ivanov et al. (2006) used a linear dependence of risk on dose as did Kreuzer et al. (2010) in their analysis of cancer and cardiovascular diseases in the German uranium miners cohort study. Non-cancer mortality was analysed in a large cohort of employees in the UK nuclear industry by McGeoghegan et al. (2008) using the following model for ERR: *R*_(*b, a, r, i, s*)_ = λ_(*b, a, r, i, s*)_[1 + *ERR*(*d*)]. Here, *R* is the cause-specific mortality rate and λ is the background mortality rate in the absence of any effects from radiation exposure. The subscripts *b*, *a*, *r*, *i*, and *s* refer respectively to birth cohort, attained age, radiation exposure status, employment status, and site of employment.* ERR*(*d*) is a function of lagged cumulative external dose (*d*) describing the excess relative risk (McGeoghegan et al. 2008). Muirhead et al. (2009) performed the latest analysis of the UK National Registry for Radiation Workers comprising a total number of 174,541 persons. They analysed among other biological endpoints the mortality from all circulatory diseases by modelling the ERR as a linear function of dose. In their analysis of the associations between low-level exposure and mortality (including mortality from ischaemic heart disease) among workers at Oak Ridge National Laboratory Richardson and Wing (1999) applied a relative risk model of the form λ(*Z*, *z*, *y*) = exp(*Z*α + β*x* + δ*y*), where the mortality rate (λ) was considered in terms of a vector of covariates (*Z*), the radiation dose accumulated before age 45 (*x*), and the radiation dose accumulated after age 45 (*y*). This is a generalized linear model. In the IARC 15-country study of radiation workers, Vrijheid et al. (2007) found increasing trends with dose for some biological endpoints and decreasing trends for others, although none were statistically significant. In that context, we point out that Vrijheid et al. (2007) based their analyses on a linear relative risk Poisson model, in which the relative risk is of the form 1 + β*Z*, where *Z* is the lagged cumulative dose in Sv and β is the excess relative risk per Sievert. Vrijheid et al. (2007) state that this model has been used commonly in analyses of nuclear workers studies and radiation risk estimation, and reference ICRP (1991) and US NRC (2006). Detailed results for the ERR found within these eight occupational studies have been summarized by Little et al. (2010). Talbott et al. (2003) reported a decreasing trend in heart disease mortality with dose for men and women exposed as a result of the accident at the Three Mile Island nuclear power station. For women, the decreasing trend was significant. The authors performed logistic regression fitting multiplicative relative risk models of the form λ(*t*) = λ_0_(*t*)exp(*x*(*t*)β) (i.e. a generalized linear model) to the cohort rates (Talbott et al. 2003). This comprises the 14 studies reviewed by Little et al. (2010) including the study on environmental exposure by Talbott et al. (2003). The authors of the current study are convinced that dose–response analyses and related risk estimations should not be based on the application of only one model (for which usually a linear increase of risk with increasing dose is assumed) unless this one model is clearly preferred by model selection techniques. In the present study, it has been demonstrated that the use of a large variety of dose–response curves leads to a better and more realistic description of dose–response curves for non-cancer vascular diseases than the use of LNT models.

MMI is a form of Bayesian model averaging (BMA; Hoeting et al. 1999). It can be shown that the formula used to perform BMA (Eq. 1 in Hoeting et al. 1999) reduces to (2) for the Akaike weights *p*_*m*_ when one assumes that a priori all models are equally likely. This is the approach chosen here with respect to the models shown in Fig. 1. The present study did not aim to find the true model but the one which fits the data best. In this case, Burnham and Anderson (2002) (p. 77) argue for equal model priors (i.e. equal prior probabilities for the models to be tested) under a so-called information-theoretic approach. A recent criticism by Richardson and Cole (2012) of applying the MMI technique in radiation epidemiology has been answered by Walsh et al. (2011).

The present study showed that the application of the MMI technique to non-cancer data of Report 13 on the atomic bomb survivors leads to distinctly nonlinear dose–response curves and related threshold-doses. This provides strong evidence that low and medium doses of ionizing radiation may have different effects than high doses. Such findings may stimulate the development of mechanistic models, which explain dose–responses based on radiobiological cellular processes. Biologically based mechanistic models are important for estimating at which stages of the disease process radiation may act (see, for example, the work of Little et al. (2009)). Motivated by the results of the present analysis, it is promising to include into mathematical models biological mechanisms (such as, for example, possible anti-inflammatory effects of low and medium doses of ionizing radiations) that may lead to distinct nonlinearities in the related dose–response curves. How this works for the biological endpoint of cancer induction after exposure to low doses of ionizing radiation at low dose rates has been shown by Schöllnberger et al. (2004, 2005) using deterministic and stochastic multi-stage models with clonal expansion.

## Conclusions

Summarizing, it can be said that the present analyses of the non-cancer mortality data from Report 13 on the atomic bomb survivors predict a strongly reduced risk for death from CVD and cardiovascular diseases excluding CVD due to ionizing radiation. For CVD, MMI yielded a weak dose–response (with a risk estimate of about one-third of the LNT model) below a step at 0.6 Gy and a stronger dose–response at higher doses. Based on 90% confidence intervals, the calculated risk estimates are consistent with zero risk below this threshold-dose. For mortalities related to cardiovascular diseases excluding CVD, an LNT-type dose–response was found with risk estimates consistent with zero risk below 2.2 Gy based on 90% confidence intervals. Great care must be taken when analysing the shape of dose–responses for non-cancer mortalities. In addition to LNT and linear threshold models, other dose–responses must also be considered and tested. Non-standard dose–response curves derived from the rigorous application of a statistical protocol may stimulate the development of mechanistic models that explain dose–responses based on radiobiological cellular processes. Analysing the shape of dose–responses by testing a series of different empirical models, as it has been done in the present study using MMI, provides valuable information for the mechanistic modelling. In practical radiation protection, MMI is an important tool for risk assessment, especially at low doses. It allows different models to be combined, leading to a more comprehensive characterization of the uncertainty of risk estimates. This conclusion also holds for other detrimental health effects such as cancer.

## Electronic supplementary material

Below is the link to the electronic supplementary material.

Supplementary material: Section 1: contains the exact form of Preston’s baseline model as it was used by Preston et al. (2003). Section 2: description of the streamlined baseline models for CVD and cardiovascular diseases. Section 3: description of the three dose-effect modifiers (sex, age at exposure, and age attained) and how they were implemented mathematically; a numerical example is given of how a dose-effect modifier influences the total hazard function *h*. Section 4: the formula applied in Poisson regression is explained. Section 5: we explain how the excess relative risk and the excess absolute risk are calculated from ERR and EAR models. Section 6: a formula is derived that can be used to calculate the city-averaged *EAR*-values for men (i.e. the *EAR*-values are than valid for men in Hiroshima and Nagasaki); the formula is to be applied to the *EAR*-values given in Table 2. Section 7: here, we explain why the categorical model fits and the fit with the Gompertz model were not included for the calculations of the MMI. Table S1 provides the results from fitting the final three non-nested models to the joint data for CVD in men and women: for all model parameters (i.e. baseline- and radiation-related parameters), the best estimates are given together with Wald-type standard errors. Analogous, Table S2 provides the results from fitting the final four non-nested models to the joint data for cardiovascular diseases in men and women: for all model parameters the best estimates are given together with Wald-type standard errors. Tables S3 and S4 provide the final deviances for mortality from CVD in men and women, respectively, analysed with the ERR-step model. The data were grouped into four dose categories and five age categories. Table S5 provides analogous results for mortality from cardiovascular diseases in women. (PDF 117 kb)

## Footnotes

1 It is noted that Preston et al. (2003) and Shimizu et al. (2010) refer to the ICD-9 codes 430–438 as “stroke”, while stroke is in fact a subgroup of ICD-9 430–438. The latter represents cerebrovascular disease.

2 It is noted that Preston et al. (2003) and Shimizu et al. (2010) refer to the ICD-9 codes 390–429, 440–459 as “heart disease”, while these ICD-9 codes are better described as cardiovascular diseases excluding CVD (Dr. Frauke Neff, Helmholtz Zentrum München, personal communication).
