# Supplementary material for: Dose–responses from multi-model inference for the non-cancer disease mortality of atomic bomb survivors
Source: Radiat Environ Biophys. 2012 Mar 22;51(2):165–78. doi: 10.1007/s00411-012-0410-4 (PMC3332375; doi:10.1007/s00411-012-0410-4)
Supplement: Supplementary file 1 — Supplementary material: Section 1: contains the exact form of Preston’s baseline model as it was used by Preston et al. (2003). Section 2: description of the streamlined baseline models for CVD and cardiovascular diseases. Section 3: description of the three dose-effect modifiers (sex, age at exposure, and age attained) and how they were implemented mathematically; a numerical example is given of how a dose-effect modifier influences the total hazard function h. Section 4: the formula applied in Poisson regression is explained. Section 5: we explain how the excess relative risk and the excess absolute risk are calculated from ERR and EAR models. Section 6: a formula is derived that can be used to calculate the city-averaged EAR-values for men (i.e. the EAR-values are than valid for men in Hiroshima and Nagasaki); the formula is to be applied to the EAR-values given in Table 2. Section 7: here, we explain why the categorical model fits and the fit with the Gompertz model were not included for the calculations of the MMI. Table S1 provides the results from fitting the final three non-nested models to the joint data for CVD in men and women: for all model parameters (i.e. baseline- and radiation-related parameters), the best estimates are given together with Wald-type standard errors. Analogous, Table S2 provides the results from fitting the final four non-nested models to the joint data for cardiovascular diseases in men and women: for all model parameters the best estimates are given together with Wald-type standard errors. Tables S3 and S4 provide the final deviances for mortality from CVD in men and women, respectively, analysed with the ERR-step model. The data were grouped into four dose categories and five age categories. Table S5 provides analogous results for mortality from cardiovascular diseases in women. (PDF 117 kb) [file 411_2012_410_MOESM1_ESM.pdf]

## Suppplementary Material

# Dose-Responses from Multi-Model Inference for the Non-cancer Disease Mortality of Atomic Bomb Survivors

H. Schöllnberger (✉), J.C. Kaiser, L. Walsh, P. Jacob

Helmholtz Zentrum München, Department of Radiation Sciences, Institute of Radiation Protection, D-85764 Neuherberg, Germany
Tel: +49 89-3187-2765
Fax: +49 89-3187-3363
E-mail: schoellnberger@helmholtz-muenchen.de

## 1. Preston baseline model

The Preston baseline model is as follows:

$$h_0 = \exp\{cmH + cfH + cmN + cfN + e30l70 \times \mathrm{e30} \times \mathrm{lage70} + e30m \times \mathrm{e30} + e30f \times \mathrm{e30}$$
$$+ e30sqm \times \mathrm{e30sq} + e30sqf \times \mathrm{e30sq} + e30qspm \times \mathrm{e30qsp} + e30qspf \times \mathrm{e30qsp}$$
$$+ e50qspm \times \mathrm{e50qsp} + e50qspf \times \mathrm{e50qsp} + l70m \times \mathrm{lage70} + l70f \times \mathrm{lage70}$$
$$+ l70sqm \times \mathrm{lage70sq} + l70sqf \times \mathrm{lage70sq} + l40qspm \times \mathrm{lage40qsp} + l40qspf \times \mathrm{lage40qsp} +$$
$$+ l70qspm \times \mathrm{lage70qsp} + l70qspf \times \mathrm{lage70qsp}\} \quad \text{(A1)}$$

with e30 := ($e$ – 30)/10, e30sq := e30 × e30, e50 := ($e$ – 50)/10,

lage70 := ln($a$/70), lage70sq := ln($a$/70) × ln($a$/70),

e30qsp := [e30] × [e30] for $e > 30$,

e50qsp := [e50] × [e50] for $e > 50$,

lage40qsp := [ln($a$/40)] × [ln($a$/40)] for $a \geq 40$,

lage70qsp := [ln($a$/70)] × [ln($a$/70)] for $a \geq 70$.

These naming conventions are taken from R13models.log. Age at exposure is denoted by *e*, age attained by *a*. Model parameters in Eq. (A1) are italicised. Parameters *cmH*, *cfH*, *cmN*, and *cfN* represent constant factors (*cmH* and *cfH*, for example, are constants related to males and females in Hiroshima), parameter *e30l70* describes variations of the hazard with multiplicative effects of age attained and age at exposure, while parameters *e30m* and *e30f* describe variations of the hazard with age at exposure for males and females, respectively. Parameters *e30qspm* and *e30qspf* mark

the dependence on a quadratic spline function with age knot at $e$ = 30 years for males and females, respectively. The Preston baseline model therefore uses 29 model parameters including the four age knots in e30qsp, e50qsp, lage40qsp, and lage70qsp.

## 2. Streamlined baseline models

One common (i.e. joint) baseline model was used for the cerebrovascular disease (CVD) data. Our streamlined baseline model for CVD contains 21 statistically significant baseline parameters - 8 parameters less than Preston's baseline model $h_0$ from Eq. (A1): *e30sqm* = *e30qspm* = *e50qspm* = *e50qspf* = *l70f* = 0 which made the three age knots related to *e30qspm*, *e50qspm*, and *e50qspf* obsolete. In other words: for the fit of the CVD data one single joint baseline model was used, namely Eq. (A1) with *e30sqm* = *e30qspm* = *e50qspm* = *e50qspf* = *l70f* = 0. In addition, it was found that the model fit significantly improved when the age knots at 40 and 70 years in lage40qsp and lage70qsp, respectively, were allowed to be free. Note that for reasons of clarity the related adjustable parameters are denoted by *l40agem*, *l40agef*, *l70agem*, and *l70agef* although best estimates different than 40 or 70 were found in the related model fits (Table S1). The fit also significantly improved when the age at exposure knot at 30 years in e30qsp was allowed to be free; the related model parameter is denoted by *e30agef*.

For cardiovascular diseases, we proceeded analogously as for CVD. Each of the 29 parameters of the Preston baseline model was tested for its significance resulting in a streamlined baseline model with 14 model parameters less than the Preston baseline model: *e30qspm* = *e30qspf* = *e50qspm* = *e50qspf* = *l70m* = *l70sqf* = *l70qspm* = 0 with the five age knots related to *e30qspm*, *e30qspf*, *e50qspm*, *e50qspf*, and *l70qspm* obsolete. In other words: for the fit of the data for cardiovascular diseases one single joint baseline model was used, namely Eq. (A1) with *e30qspm* = *e30qspf* = *e50qspm* = *e50qspf* = *l70m* = *l70sqf* = *l70qspm* = 0. Furthermore, it was found that for cardiovascular diseases there was no statistically significant city effect: instead of the four baseline parameters *cmH*, *cfH*, *cmN*, and *cfN* applied for CVD (Table S1) only two remain for cardiovascular diseases: *cm* and *cf* (Table S2). The streamlined baseline model for cardiovascular diseases therefore has 15 (29 – 12 – 2) model parameters (see

Table S2 in the Online Resource). In addition, it was found that the model fit significantly improved when the age knots at 40 and 70 in lage40qsp and lage70qsp, respectively, were allowed to be free (Table S2).

**3. Dose-effect modifiers**

Three dose-effect modifiers were implemented into the various risk models. For an excess relative risk (ERR) model, the following form was applied: $h = h_0 \times (1 + ERR(D, s, a, e))$ where $D$, $s$, $a$, $e$ stand for dose, sex, attained age and age at exposure, respectively. It is $ERR(D, s, a, e) = err(D) \times \exp(dem_1 \times \text{sex} + dem_2 \times \text{e30} + dem_3 \times \text{lage70})$. Here, $err(D)$ is any of the dose-responses depicted in Fig. 1. The general form of an excess absolute risk (EAR) model is $h = h_0 + EAR(D, s, a, e)$ where $EAR(D, s, a, e) = ear(D) \times \exp(dem_1 \times \text{sex} + dem_2 \times \text{e30} + dem_3 \times \text{lage70})$ and $ear(D)$ is any of the dose-responses from Fig. 1. Here, $dem_1$, $dem_2$, and $dem_3$ are three adjustable parameters related to the three dose-effect modifiers sex, age at exposure, and age attained. The naming conventions for e30 and lage70 are provided after Eq. (A1).

When fitting the mortality data for cardiovascular diseases we found for the EAR-LNT model and the EAR-quadratic model that age was a statistically significant dose effect modifier with the related parameter *dem3* = 5.1 (Table S2). That gives the factor $\exp(5.1 \times \ln(a/70))$. At an attained age of $a = 56$ years, the mean age attained of all individuals registered within the data set for cardiovascular diseases, we therefore have $h = h_0 + 0.29 \times ear(D)$. For $a = 70$ years we simply have $h = h_0 + ear(D)$. Consequently, for lower ages the *EAR* (shown in Fig. 3 for $a = 70$ years) calculated with the EAR-LNT model is strongly decreased. This explains the seemingly inconsistent shape of the *EAR* for the EAR-LNT model in Fig. 3. Because of the relatively large weight of the EAR-LNT model (0.3619; see Table 1), the *EAR* for the MMI is then also shifted towards lower values.

For all other model fits no significant effect modifiers were found (Tables S1 and S2).

## 4. Poisson regression

The MECAN software (Kaiser 2010) uses the maximum likelihood method to estimate the values of the adjustable model parameters by fitting the model to the data. Because maximizing the likelihood is equivalent to minimizing the –ln(Likelihood), the latter problem, which is numerically better tractable, is solved in MECAN to find the best model solution. For grouped person-year data such as the grouped LSS data, the likelihood corresponding to a Poisson model is used: $-\ln(L) = \sum_i \left[\Lambda_i - n_i + n_i \ln\left(n_i / \Lambda_i\right)\right]$ where $n_i$ is the observed number of cases (i.e. the number of fatal CVDs or cardiovascular diseases) in group *i* and $\Lambda_i$ is the calculated (expected) number of cases in group *i*. The deviance is defined as *dev* := –2 × ln(MaxLikelihood).

## 5. *ERR* and *EAR* calculated from different models

For CVD, we found that two different ERR models are preferable (Table 1). The general form of an ERR model is $h = h_0 \times RR = h_0 \times (1 + ERR)$, where *RR* is the relative risk. Consequently, when we calculate the *EAR* from an ERR model we get $EAR = h - h_0 = h_0 \times (1 + ERR) - h_0 = h_0 \times ERR$. For CVD, the baseline model, $h_0$, depends on city and sex via model parameters *cmH, cfH, cmN*, and *cfN* (Table S1). Therefore, the *EAR*-values for CVD (Table 2) also depend on city and sex: they are only valid for males from Hiroshima, as stated in the *Results* section of the main text. When the *ERR* is calculated from an ERR model, then only the shape of the dose-responses related to the excess risk from radiation enters the risk estimate. Fig. 2 is therefore valid for males and females from both cities.

For cardiovascular diseases, it was found that three different EAR *models* are preferable (Table 1). The general form of the EAR model is $h = h_0 + EAR$. When the *ERR* is calculated from an EAR model, one gets $ERR = h/h_0 - 1 = (h_0 + EAR)/h_0 - 1 = EAR/h_0$. For cardiovascular diseases, we found that $h_0$ is dependent on sex (but not city) via model parameters *cm* and *cf* (Table S2). Therefore, the *ERR*-values for cardiovascular diseases

(Table 3) also depend on sex: they are only valid for males. Related to Fig. 3 it can be said that the *EAR* calculated from an EAR model only depends on the shapes of the preferable dose-responses for the excess risk from radiation. Therefore, Fig. 3 is valid for males and females from both cities.

## 6. Derivation of city-averaged *EAR*-values to be used in Table 2

In the previous section it has been derived that for CVD the *EAR* can be calculated from an ERR model as follows: $EAR = h_0 \times ERR$. The city-averaged EAR-values for males, $\langle EAR \rangle_m$, can be calculated as follows:

$$\langle EAR \rangle_m = w_H \times EAR_H + w_N \times EAR_N = ERR \times (w_H \times h_{0,mH} + w_N \times h_{0,mN}). \qquad \text{(A2)}$$

Here, $w_H = \mathrm{PY}_H/\mathrm{PY}_{tot}$ is the number of person years in Hiroshima divided by the total number of person years in the data set; $w_N = \mathrm{PY}_N/\mathrm{PY}_{tot}$ is the number of person years in Nagasaki divided by the total number of person years in the data set, and $h_{0,mH}$ is the streamlined baseline hazard for males in Hiroshima, i.e. Eq. (A1) with the numerical values for the model parameters taken from Table S1 using $e30sqm = e30qspm = e50qspm = e50qspf = l70f = 0$ (refer to section 2 above) and with $cfH = cmN = cfN = 0$. An analogous definition holds for $\mathrm{h}_{0,mN}$. For the ERR-LNT model we therefore have

$h_{0,mH}$ = exp{–9.57 + 0.44 × e30 × lage70 + 0.504 × e30 + 2.07 × e30 + 0.522 × e30sq – 0.581 × e30qsp – 11.32 × lage70 – 14.3 × lage70sq – 5.6 × lage70sq + 16.33 × lage40qsp + 8.9 × lage40qsp – 120 × lage70qsp – 134 × lage70qsp} = exp{–9.57} × exp{*rest*} where *rest* stands for all other terms in the exponential function.

The right hand side of Eq. (A2) can be expanded as follows:

$$ERR \times (w_H \times h_{0,mH} + w_H \times h_{0,mN}) = ERR \times \ h_{0,mH} \times \left( w_H + w_N \frac{h_{0,mN}}{h_{0,mH}} \right). \tag{A3}$$

With Eq. (A1) one finds

$$ERR \times \ h_{0,mH} \times \left( w_H + w_N \frac{h_{0,mN}}{h_{0,mH}} \right) = \ ERR \times \ h_{0,mH} \times \left( w_H + w_N \frac{e^{cmN}}{e^{cmH}} \right) =$$

$$ERR \times \ h_{0,mH} \times \left( w_H + w_N e^{(cmN - cmH)} \right). \tag{A4}$$

Considering Eq. (A2) and using $EAR = h_0 \times ERR$ within Eq. (A4), one obtains

$$\langle EAR \rangle_m = EAR_{mH} \times \left( w_H + w_N e^{(cmN - cmH)} \right). \tag{A5}$$

The term $EAR_{mH}$ stands for the *EAR*-values for males in Hiroshima as given in Table 2. The city-averaged *EAR*-values for males can be obtained by multiplication with the correction factor of $\left( w_H + w_N e^{(cmN - cmH)} \right)$. It is $w_H$ = $PY_H/PY_{tot}$ = 818938.31/1200991.78 = 0.68 and $w_N$ = $PY_N/PY_{tot}$ = 382053.47/1200991.78 = 0.32. Using the best estimates from Table S2, we find that for all three preferred models the correction factor is 1.1. Applying the *AIC*-weights from Table 1 therefore yields again the correction factor of 1.1, which can be applied to calculate from the *EAR*-values

for MMI in Table 2 the city-averaged *EAR*-values (for example: for the MMI the *EAR*-value at 1 Gy for males in Hiroshima with an age attained of 70 years, exposed at an age of 30 years, is 6.6 per $10^4$ PY; therefore, the city-averaged *EAR*-value for males is 6.6 × 1.1 = 7.3 per $10^4$ PY. This risk prediction holds for males in Hiroshima and Nagasaki).

## 7. Neglection of categorical and Gompertz models for MMI

It is noted that the categorical model (#11 in Fig. 1) is a non-nested model like those summarized in Table 1. It was, however, not used for MMI because of its very small contributions to the *AIC*-weights. The categorical model fit to the data for CVD yielded $\Delta AIC = 9.85$ (with $dev = 3565.9$ and $Npar = 28$). Using Eq. (2) with $n = 2$ and $m = 1$ (i.e. comparing with the best model, which has $\Delta AIC = 0$) one obtains $p_1 = \exp(-\Delta AIC_1/2)/(1+\exp(-\Delta AIC_1/2)) = 0.0072$. For cardiovascular diseases we found $\Delta AIC = 10.65$ (with $dev = 3694.38$, $Npar = 22$). That gives $p_1 = 0.0048$. Both values are far below the threshold value of 0.05 that is used as a cut-off in the scientific literature (Hoeting et al. 1999, Walsh 2007). The fit of the data for cardiovascular diseases with the Gompertz function led to $p_1 = 0.035$; therefore this model was not used for MMI either.

## References


Hoeting JA, Madigan D, Raftery AE, Volinsky CT (1999) Bayesian Model Averaging: A Tutorial Statist Sci 14:382-417

Kaiser JC (2010) MECAN. A Software Package to Estimate Health Risks in Radiation Epidemiology with Multi-Model Inference. User Manual. Version 0.2 (Helmholtz Zentrum München, Neuherberg, Germany)

Walsh L (2007) A short review of model selection techniques for radiation epidemiology. Radiat Environ Biophys 46:205-213

**Table S1** Results from fitting the final three non-nested models to the joint mortality data for CVD in males and females: Best estimates and Wald-type standard errors (in parentheses) for the model parameters of the three preferable model fits for CVD. The numbers in brackets refer to the eleven dose-responses depicted in Fig. 1 in the main text. Parameters 1 to 21 are the baseline parameters, parameters 22 and 23 are radiation related parameters, i.e. they refer to the risk models #1, #2, and #6 depicted in Fig. 1.

| | Parameter | ERR-LNT model [#1] | ERR-quadratic model [#2] | ERR-step model[a] [#6] |
|---|---|---|---|---|
| 1 | *cmH* | -9.57 (0.23) | -9.56 (0.24) | -9.573 (0.030) |
| 2 | *cfH* | -5.96 (0.14) | -5.96 (0.13) | -5.937 (0.029) |
| 3 | *cmN* | -9.40 (0.23) | -9.38 (0.25) | -9.404 (0.050) |
| 4 | *cfN* | -5.96 (0.14) | -5.96 (0.13) | -5.945 (0.044) |
| 5 | *e30l70* ($yr^{-1}$) | 0.44 (0.18) | 0.44 (0.18) | 0.447 (0.043) |
| 6 | *e30m* ($yr^{-1}$) | 0.504 (0.034) | 0.504 (0.034) | 0.505 (0.014) |
| 7 | *e30f* ($yr^{-1}$) | 2.07 (0.11) | 2.06 (0.12) | 2.083 (0.012) |
| 8 | *e30sqf* ($yr^{-2}$) | 0.522 (0.032) | 0.520 (0.033) | 0.5259 (0.0060) |
| 9 | *e30qspf* ($yr^{-2}$) | -0.581 (0.025) | -0.578 (0.026) | -0.5850 (0.0022) |
| 10 | *l70m* | -11.32 (0.80) | -11.32 (0.85) | -11.33 (0.14) |
| 11 | *l70sqm* | -14.3 (1.2) | -14.3 (1.3) | -14.32 (0.42) |
| 12 | *l70sqf* | -5.6 (1.2) | -5.6 (1.2) | -5.65 (0.45) |
| 13 | *l40qspm* | 16.33 (0.73) | 16.33 (0.79) | 16.314 (0.065) |
| 14 | *l40qspf* | 8.9 (2.1) | 8.9 (2.1) | 9.01 (0.17) |
| 15 | *l70qspm* | -120 (49) | -120 (49) | -119 (16) |
| 16 | *l70qspf* | -134 (47) | -134 (47) | -135 (50) |
| 17 | *l40agem*[b] (yr) | 42.57 (0.49) | 42.57 (0.49) | 42.537 (0.055) |
| 18 | *l40agef* (yr) | 51.3 (3.8) | 51.3 (3.8) | 51.44 (0.14) |
| 19 | *l70agem*[c] (yr) | 84.0 (1.2) | 84.0 (1.2) | 83.98 (0.36) |
| 20 | *l70agef* (yr) | 85.5 (1.0) | 85.5 (1.0) | 85.5 (1.0) |
| 21 | *e30agef*[d] (yr) | 17.88 (0.80) | 17.91 (0.88) | 17.866 (0.061) |
| 22 | | *err* = 0.124 $Gy^{-1}$ (0.059) | *err* = 0.063 $Gy^{-2}$ (0.033) | *scale* = 0.218 (0.076) |
| 23 | | | | $D_{th}$ = 0.620 Gy (< 1%) |
| | | | | |
| | deviance | 3569.51 | 3570.14 | 3566.05 |

[a] Implemented as a hyperbolic tangent function with a fixed value for the slope *s*: $10^5$/Gy. This parameter does not count as a model parameter because $1/s \approx 0$ compared to all other parameter values.

[b] Denotes the age knot for males in lage40qsp (refer to the naming conventions after Eq. (A1)).

[c] Denotes the age knot for males in lage70qsp.

[d] Denotes the age at exposure knot for females in e30qsp.

**Table S2** Results from fitting the final four non-nested models to the joint mortality data for cardiovascular diseases in males and females: Best estimates and Wald-type standard errors (in parentheses) for the model parameters of the four preferable model fits for cardiovascular diseases. The numbers in brackets refer to the eleven dose-responses depicted in Fig. 1 in the main text. Parameters 1 to 15 are the baseline parameters, parameters 16 and 17 are radiation related parameters, i.e. they refer to the risk models #1, #2, #5, and #6 depicted in Fig. 1.

| | Parameter | EAR-LNT model [#1] | EAR-quadratic model [#2] | EAR-threshold model [#5] | EAR-step model[b] [#6] |
|---|---|---|---|---|---|
| 1 | *cm*[a] | -6.52 (0.12) | -6.52 (0.12) | -6.51 (0.11) | -6.52 (0.12) |
| 2 | *cf* | -8.26 (0.17) | -8.12 (0.17) | -8.07 (0.17) | -8.06 (0.17) |
| 3 | *e30l70* ($yr^{-1}$) | 0.87 (0.17) | 0.87 (0.18) | 0.86 (0.17) | 0.86 (0.17) |
| 4 | *e30m* ($yr^{-1}$) | 0.267 (0.037) | 0.265 (0.037) | 0.264 (0.036) | 0.263 (0.036) |
| 5 | *e30f* ($yr^{-1}$) | 0.446 (0.040) | 0.435 (0.039) | 0.429 (0.038) | 0.429 (0.038) |
| 6 | *e30sqm* ($yr^{-2}$) | -0.057 (0.017) | -0.057 (0.017) | -0.056 (0.017) | -0.056 (0.017) |
| 7 | *e30sqf* ($yr^{-2}$) | -0.075 (0.018) | -0.073 (0.019) | -0.070 (0.018) | -0.070 (0.018) |
| 8 | *l70f* | 1.89 (0.38) | 2.12 (0.38) | 2.20 (0.37) | 2.23 (0.37) |
| 9 | *l70sqm* | -5.43 (0.75) | -5.40 (0.76) | -5.36 (0.74) | -5.36 (0.74) |
| 10 | *l40qspm* | 7.98 (0.59) | 7.87 (0.59) | 7.79 (0.57) | 7.76 (0.57) |
| 11 | *l40qspf* | 3.04 (0.18) | 2.90 (0.18) | 2.82 (0.18) | 2.80 (0.18) |
| 12 | *l70qspf* | -139 (49) | 140 (49) | -141 (50) | -141 (50) |
| 13 | *l40agem*[c] (yr) | 47.1 (1.3) | 46.9 (1.3) | 46.7 (1.3) | 46.6 (1.3) |
| 14 | *l40agef* (yr) | 28.71 (0.83) | 28.70 (0.88) | 28.55 (0.88) | 28.54 (0.88) |
| 15 | *l70agef*[d] (yr) | 86.39 (0.86) | 86.44 (0.89) | 86.52 (0.86) | 86.52 (0.86) |
| 16 | | $ear = 8.8 \cdot 10^{-4}$ $Gy^{-1}$ $pyr^{-1}$ ($2.9 \cdot 10^{-4}$) | $ear = 3.9 \cdot 10^{-4}$ $Gy^{-2}$ $pyr^{-1}$ ($1.7 \cdot 10^{-4}$) | $ear = 2.1 \cdot 10^{-3}$ $Gy^{-1}$ $pyr^{-1}$ (0.0013) | $scale = 1.16 \cdot 10^{-3}$ $pyr^{-1}$ ($5.0 \cdot 10^{-4}$) |
| 17 | | $dem_3$ = 5.1 (1.4) | 4.1 (1.6) | $D_{th}$ = 2.01 Gy (0.26) | $D_{th}$ = 2.194 Gy (< 1%) |
| | | | | | |
| | deviance | 3693.73 | 3694.05 | 3695.0 | 3695.66 |

[a] Parameter *cm* is a constant related to males.

[b] Implemented as a hyperbolic tangent function with a fixed value for the slope *s*: $10^5$/Gy. This parameter does not count as a model parameter because $1/s \approx 0$ compared to all other parameter values.

[c] Denotes the age knot for males in lage40qsp (refer to the naming conventions after Eq. (A1)).

[d] Denotes the age knot for females in lage70qsp.

**Table S3** Final deviances for CVD in males analyzed with ERR-step model: Final deviances obtained by forward calculations using the ERR-step model (with $D_{th}$ = 0.62 Gy) in combination with the mortality data for CVD in males. For the calculations the best estimates from Table S1 were applied. Data were grouped into four dose categories and five age categories. As a comparison, the deviances are also shown for Preston's ERR-LNT model.

| | **0-20 yr** | **20-40 yr** | **40-60 yr** | **60-80 yr** | **80 yr -** | **sum** | **Preston's ERR-LNT model** |
|---|---|---|---|---|---|---|---|
| ***D* ≤ 0.005 Gy** | 155.79 | | | | | 155.79 | 154.33 |
| **0.005 < *D* ≤ 0.1 Gy** | 652.09 | | | | | 652.09 | 651.0 |
| **0.1 < *D* ≤ 0.5 Gy** | 0 | 9.36 | 106.47 | 256.02 | 150.34 | 522.19 | 530.52 |
| ***D* > 0.5 Gy** | 449.57 | | | | | 449.57 | 452.47 |

**Table S4** Final deviances for CVD in females analyzed with ERR-step model: Final deviances obtained by forward calculations using the ERR-step model (with $D_{th}$ = 0.62 Gy) in combination with the mortality data for CVD in females. For the calculations the best estimates from Table S1 were applied. Data were grouped into five dose categories and five age categories. As a comparison, the deviances are also shown for Preston's ERR-LNT model.

| | **0-20 yr** | **20-40 yr** | **40-60 yr** | **60-80 yr** | **80 yr -** | **sum** | **Preston's ERR-LNT model** |
|---|---|---|---|---|---|---|---|
| ***D* ≤ 0.005 Gy** | | | 149.09 | | | 149.09 | 149.90 |
| **0.005 < *D* ≤ 0.1 Gy** | 0 | 13.73 | 131.23 | 273.93 | 168.23 | 587.12 | 597.72 |
| **0.1 < *D* ≤ 0.5 Gy** | | | 623.38 | | | 623.38 | 626.25 |
| **0.5 < *D* ≤ 1 Gy** | 0 | 0.54 | 45.98 | 96.95 | 58.0 | 201.48 | 210.72 |
| ***D* > 1 Gy** | | | 225.35 | | | 225.35 | 226.66 |

**Table S5** Final deviances for cardiovascular diseases in females analyzed with EAR-LNT model: Final deviances obtained by forward calculations using the EAR-LNT model in combination with the mortality data for cardiovascular diseases in females. For the calculations the best estimates from Table S2 were applied. Data were grouped into five dose categories and five age categories. As a comparison, the deviances are also shown for Preston's ERR-LNT model.

| | **0-20 yr** | **20-40 yr** | **40-60 yr** | **60-80 yr** | **80 yr -** | **sum** | **Preston's ERR-LNT model** |
|---|---|---|---|---|---|---|---|
| ***D*** **≤ 0.005 Gy** | 127.09 | | | | | 127.09 | 128.96 |
| **0.005 <** ***D*** **≤ 0.1 Gy** | 614.56 | | | | | 614.56 | 612.06 |
| **0.1 <** ***D*** **≤ 0.5 Gy** | 0 | 7.87 | 65.92 | 307.03 | 179.71 | 560.52 | 568.84 |
| **0.5 <** ***D*** **≤ 1 Gy** | 0 | 0.85 | 33.02 | 103.08 | 54.62 | 191.56 | 195.22 |
| ***D*** **> 1 Gy** | 284.79 | | | | | 284.79 | 288.76 |
